# The causal association of smoking, alcohol intake, and coffee intake with the risk of bacterial pneumonia: A Mendelian randomization study

**DOI:** 10.1097/MD.0000000000040702

**Published:** 2024-12-13

**Authors:** Zhendong He, Leting Zheng, Zhanrui Chen, Jing Wen, Fang Qin, Hanyou Mo

**Affiliations:** a Department of Rheumatology and Immunology, First Affiliated Hospital of Guangxi Medical University, Nanning, P. R. China.

**Keywords:** alcohol, bacterial pneumonia, coffee, Mendelian randomization, smoking

## Abstract

**Background:**

At present, the association of smoking, alcohol intake, and coffee intake with the risk of bacterial pneumonia (BP) remains controversial. In this study, we used a 2-sample Mendelian randomization (MR) analysis to estimate the association of smoking, alcohol intake, and coffee intake with the risk of BP.

**Methods:**

We extracted genetic variants associated with smoking initiation and cigarettes per day from the Genome-Wide Association Study and Sequencing Consortium of Alcohol and Nicotine Use database (944,625 individuals). We also extracted genetic variants associated with past tobacco smoking, alcohol intake frequency, and coffee intake from the UK Biobank database (1,316,166 individuals). BP outcomes were chosen from the FinnGen genome-wide association studies (GWAS) database (7987 patients and 188,868 controls). The inverse variance-weighted method was used primarily to calculate odds ratios (OR) and 95% confidence intervals (CI). Sensitivity analysis using different approaches such as weighted median, MR Egger, and MR pleiotropy residual sum and outlier (MR-PRESSO) have been implemented, as well as leave-one-out analysis to identify pleiotropy.

**Results:**

The 2-sample MR analysis supported the causal association of genetically predicted cigarettes per day (OR: 1.23, 95% CI: [1.08–1.39], *P* < .01] and smoking initiation (OR: 1.22, 95% CI: [1.03–1.44], *P* = .02) with the risk of BP, but not past tobacco smoking, alcohol intake frequency, and coffee intake. Heterogeneity (*P* > .05) and pleiotropy (*P* > .05) tests provided confirmatory evidence for the validity of our MR estimates.

**Conclusion:**

Our findings provide relevant evidence for a favorable causal association of genetically predicted smoking initiation and cigarettes per day with BP risk. However, there may not be a causal association between past tobacco smoking, alcohol intake, and coffee intake with increased BP incidence rates.

## 1. Introduction

A significant cause of morbidity and mortality in patients of all ages, bacterial pneumonia (BP) is the most common type of respiratory tract infection and its onset may be associated with a variety of risk factors. The risk of BP appears to be associated with factors such as drug use, environment, and lifestyle, but no controllable risk factors for BP have been identified.^[1]^

Previous studies have identified an interesting phenomenon, with some of the most prevalent lifestyles, such as smoking, alcohol intake, and coffee intake being significantly associated with the risk of BP development.^[2,3]^ Studies have shown that smoking and alcohol intake are positively associated with a poor prognosis in BP, of course, it cannot be excluded that these behaviors themselves adversely affect survival.^[4–6]^

A large prospective cohort study in the USA investigating the relationship between coffee intake and overall and cause-specific mortality showed that pneumonia and influenza deaths were negatively associated with coffee intake.^[7]^ However, due to possible residual confounders and the lack of high-quality randomized controlled trials, the associations of smoking, alcohol intake, and coffee intake with BP remain contradictory. Therefore, the existence of a causal relationship between smoking, alcohol intake, and coffee intake with BP needs to be urgently investigated.

Mendelian randomization (MR) analysis is a method for determining whether an exposure has a causal effect on an outcome.^[8]^ To reduce confounding and reverse causation in observational data, MR analysis is designed to utilize single nucleotide polymorphisms (SNP) as instrumental variants (IVs) of risk factors.^[9]^ Because MR analysis relies on the random assignment of alleles during meiosis, it is less affected by confounding factors and can reverse causality.

The 2-sample MR analysis is considered a method to identify causal relationships between exposure phenotypes and outcomes by using genetic variants of exposure as IVs, which allows the use of public datasets accessible in the genome-wide association studies (GWAS) for both “exposure” (as a risk factor) and “outcome” (as a disease), and compensates for the typical shortcomings of observational studies. Summary data from GWAS are readily available and have large datasets. This enhances the genetic interpretation of IVs at exposure and improves the accuracy and reliability of the results for 2-sample MR analysis.^[10]^

This study is a secondary data review of existing databases. In this study, we assessed the association of smoking, alcohol intake, and coffee intake with the risk of BP using a 2-sample MR analysis to determine whether these most prevalent lifestyles have a causal rather than pleiotropic effect on BP.

## 2. Materials and methods

### 2.1. Study design

**Figure 1.**
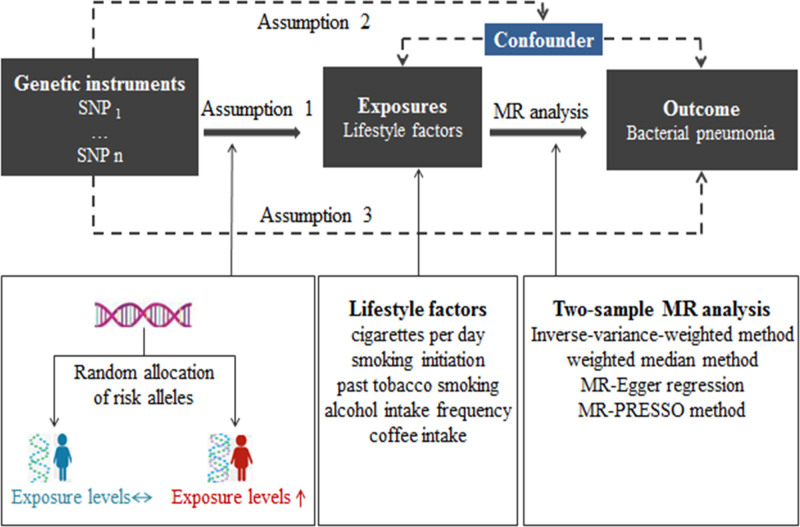
Workflow of the MR study demonstrating the link between lifestyle factors and BP. MR = Mendelian randomization, MR-PRESSO = MR Pleiotropy Residual Sum and Outlier, SNP = single nucleotide polymorphisms.

The design of this study was based on 3 assumptions: the assumption of relevance: the IVs selected were directly related to the exposure of interest; the assumption of independence: the IVs selected were not related to any confounding variables between exposure and outcome; the assumption of exclusion of limitations: the IVs selected did not affect outcome except through its correlation with exposure.^[11]^ Using 2-sample MR analysis to assess the causal relationship between lifestyle factors (exposures: smoking, alcohol intake, and coffee intake) and the risk of BP (outcome). The study design for the MR analysis is shown in Figure 1.

### 2.2. Data source

Publicly available GWAS databases were searched to obtain eligible datasets of exposure and outcomes, including GWAS and Sequencing Consortium of Alcohol and Nicotine Use, UK Biobank, and FinnGen GWAS. Therefore, no additional ethical approvals were required. As population confounding can lead to biased estimates, we restricted the genetic background of the population for the MR analysis to individuals of European ancestry.

From the GWAS and Sequencing Consortium of Alcohol and Nicotine Use database, we extracted IVs for smoking initiation and cigarettes per day. For the smoking initiation GWAS, 607,291 European individuals were included. Smoking initiation was defined as having ever been a regular smoker in life (current or former). A total of 93 SNPs associated with smoking initiation were identified (Table S1, Supplemental Digital Content, http://links.lww.com/MD/O115). For the cigarettes per day GWAS, 337,334 European individuals were included and 23 significant SNPs were identified (Table S2, Supplemental Digital Content, http://links.lww.com/MD/O115). We extracted IVs of past tobacco smoking, alcohol intake frequency, and coffee intake from the UK Biobank database. For the past tobacco-smoking GWAS, they included 424,960 Europeans and a total of 101 SNPs were significant (Table S3, Supplemental Digital Content, http://links.lww.com/MD/O115). For the alcohol intake frequency GWAS, they included 462,346 Europeans and a total of 99 SNPs were significant (Table S4, Supplemental Digital Content, http://links.lww.com/MD/O115). For the coffee intake GWAS, they included 428,860 Europeans and a total of 40 SNPs were significant (Table S5, Supplemental Digital Content, http://links.lww.com/MD/O115). The GWAS summary data for BP were extracted from the FinnGen GWAS, which included 196,855 Europeans (7987 patients and 188,868 controls). More information is available in the Supplemental Digital Content, http://links.lww.com/MD/O115.

Genetic IVs for the exposure were selected at the genome-wide significance level (*P* < 5 × 10^−8^, linkage disequilibrium *r*^2^ < 0.001) across a 1 Mb region.^[12]^ To assess the presence of weak instrument bias, we calculated estimates of the strength of the association between the genetic instruments and lifestyle factors using the *F* statistic (*F* = beta2/se^2^). This was to determine whether the instruments had sufficient explanatory power to influence the outcome variable. The *F* statistic range of the instrumental SNPs used in the MR analysis was 29.7 to 961.0, exceeding the proposed threshold of *F* > 10.^[13]^ Palindromic SNPs were removed from the instrumental variables.

### 2.3. MR analysis

The 2-sample MR analysis was used to identify genetic associations from each GWAS dataset. IVW method with random effects was used as the primary method of statistical analysis. The advantage of the random effects model is that it takes into account the variation in the effect sizes of the selected SNPs on the exposed phenotypes.^[14]^ The IVW method, which assumes that all SNPs are valid IVs and independent of each other, is used to meta-summarize the effects of different loci in MR analysis of multiple SNPs.^[15]^ To avoid the influence of unidentified and immeasurable confounders, weighted median, MR Egger, weighted mode, and simple mode were used as supplementary analysis.

### 2.4. Sensitivity analysis

Sensitivity analysis included the horizontal pleiotropy test, heterogeneity test, and the “leave-one-out” analysis. The horizontal pleiotropy test was checked by the MR Egger regression test. A significant intercept term in the MR Egger regression analysis indicates that the study has horizontal pleiotropy. Cochran *Q* values were used to estimate the heterogeneity among selected IVs for each exposure. Significant heterogeneity of analytical results was indicated if Cochran *Q* statistic test was statistically significant (*P* ≤ .05). Leave-one-out analysis was used to evaluate whether significant results were determined by a single SNP. The risk relationship between lifestyle factors (smoking, alcohol intake, and coffee intake) and BP was presented as an odds ratio (OR) and its 95% confidence interval (CI). *P* ≤ .05 was considered to indicate a possible causal relationship. All statistical analyses were performed using the TwoSampleMR, MRInstruments, and MRPROESSO packages in R software 4.0.5, and all *P* values were 2-sided.

## 3. Results

### 3.1. Association of smoking, alcohol intake, and coffee intake with the risk of BP

In the IVW method, cigarettes per day were found to be positively associated with the risk of BP (OR = 1.23, 95% CI = 1.08–1.39, *P* < .01). No horizontal pleiotropy was demonstrated in the MR Egger regression analysis (intercept *P* = .68). The association between cigarettes per day and BP remained stable in the MR Egger method (OR = 1.27, 95% CI = 1.02–1.58, *P* = .04) and weighted median method (OR = 1.27, 95% CI = 1.07–1.51, *P* = .01). Smoking initiation was positively associated with the risk of BP in the IVW method (OR = 1.22, 95% CI = 1.03–1.44, *P* = .02) and was shown to be not horizontally pleiotropic in the MR Egger regression analysis (intercept *P* = .36). There were no associations of genetic liability to past tobacco smoking, alcohol intake frequency, and coffee intake with the risk of BP.

### 3.2. Sensitivity analysis

Horizontal pleiotropy between SNPs and outcomes was assessed by MR Egger regression analysis. The MR Egger regression analysis intercept *P* > .05 for cigarettes per day (intercept *P* = .68), smoking initiation (intercept *P* = .36), past tobacco smoking (intercept *P* = .10), alcohol intake frequency (intercept *P* = .41), and coffee intake (intercept *P* = .06) showed no evidence of horizontal pleiotropy. Selected SNPs were tested for heterogeneity by Cochran test. In the IVW and MR Egger methods, Q-pval for cigarettes per day, smoking initiation, past tobacco smoking, alcohol intake frequency, and coffee intake were all greater than 0.05. The results indicated that there was no heterogeneity. No outliers were detected with the MR-PRESSO method (*P* > .05). Scatter plots of the various methods visualize that cigarettes per day are positively correlated with the risk of BP, and smoking initiation may be positively correlated with the risk of BP. The leave-one-out analysis showed that no single SNP had a dominant effect on the overall assessment. In contrast, no significant correlation was seen between past tobacco smoking, alcohol intake frequency, and coffee intake with BP.

**Figure 2.**
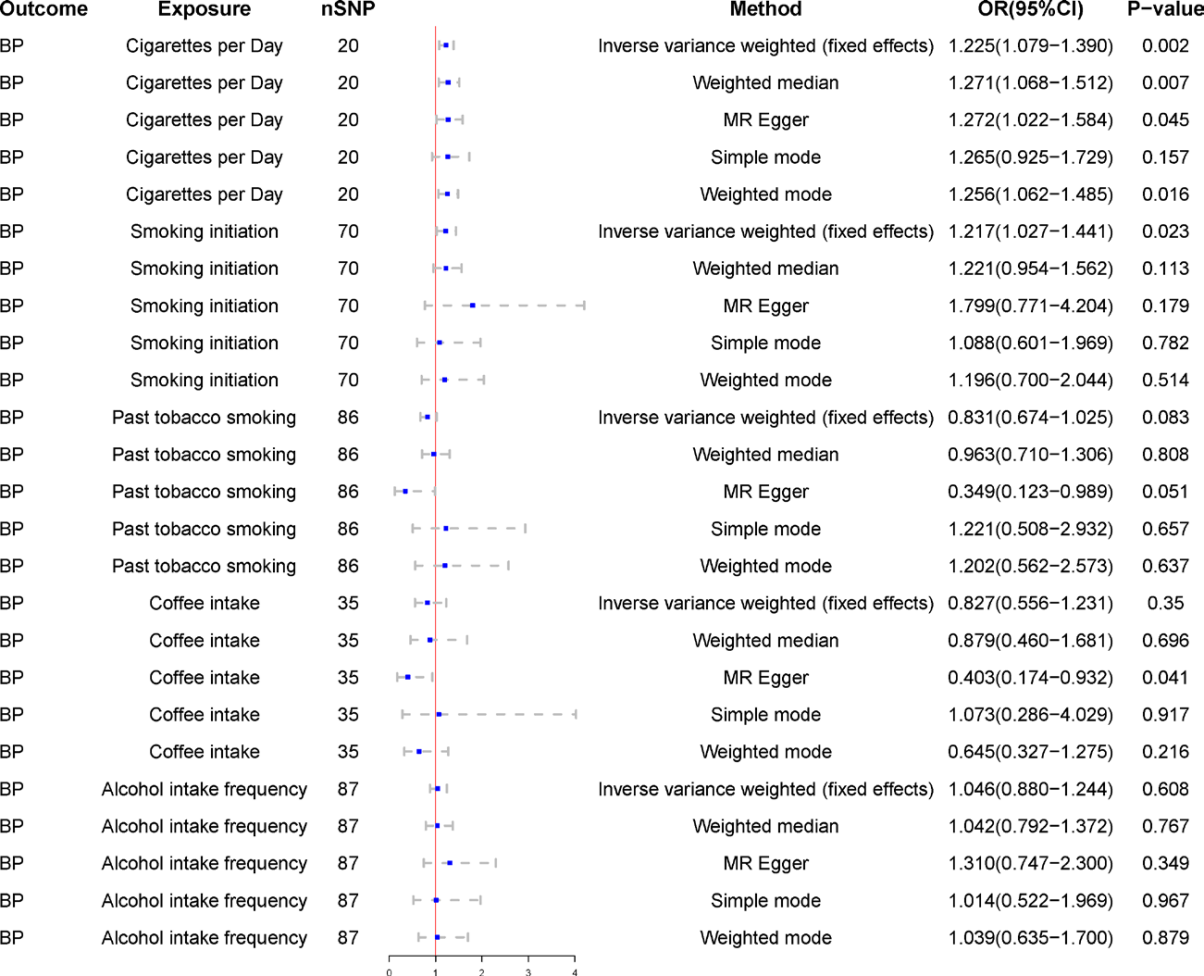
Association of smoking, alcohol intake, and coffee intake with the risk of BP in the Mendelian randomization analysis. BP = bacterial pneumonia, CI = confidence interval, MR = Mendelian randomization, OR = odds ratio, SNP = single nucleotide polymorphisms.

**Figure 3.**
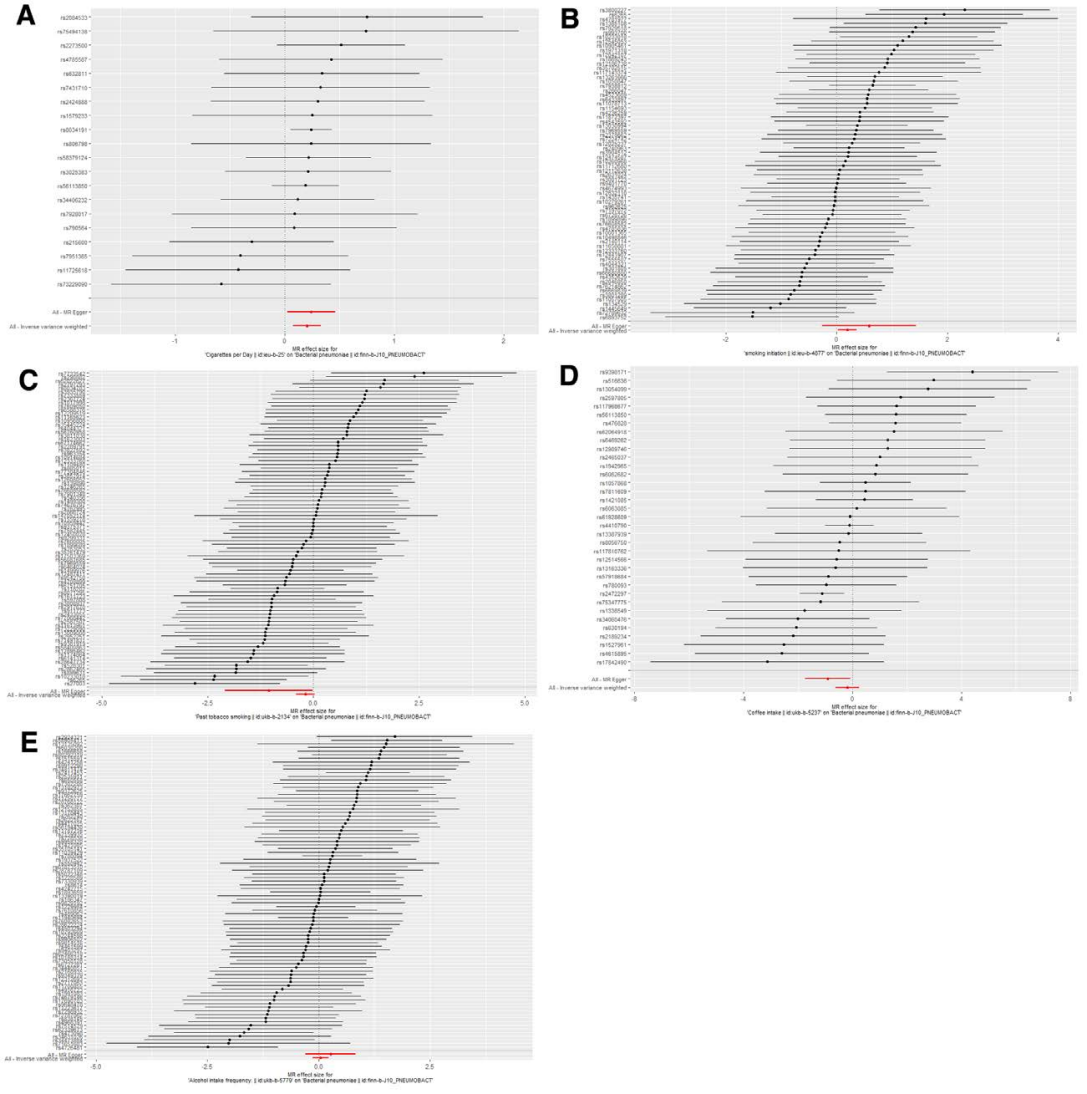
Detailed forest plots with the estimated MR effect of each IV in IVW models. (A) Cigarettes per day; (B) smoking initiation; (C) past tobacco smoking; (D) alcohol intake frequency; (E) coffee intake. IV = instrumental variant, MR = Mendelian randomization.

**Figure 4.**
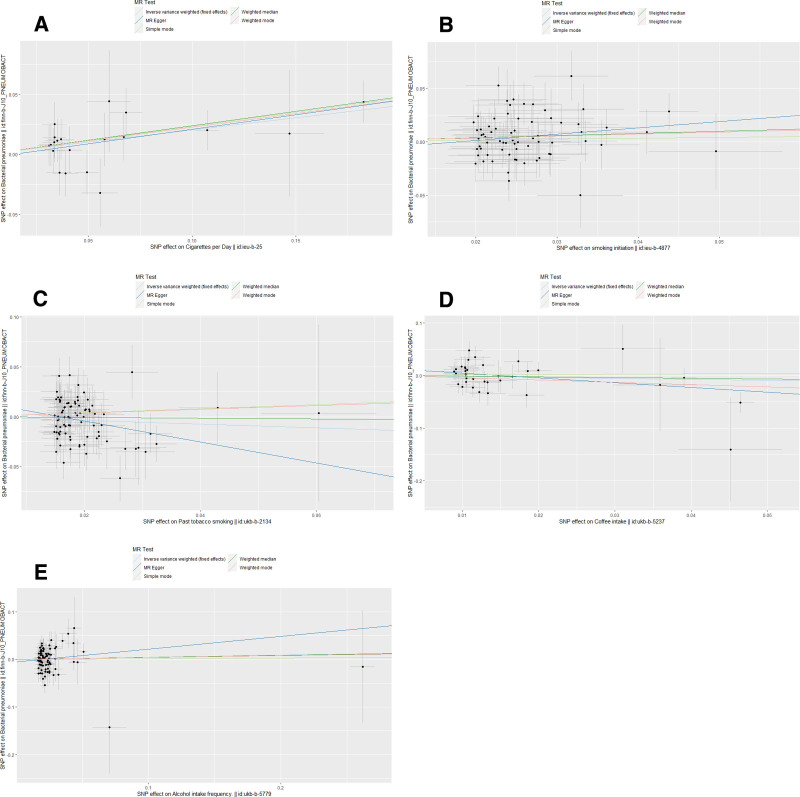
Scatter plots of causality. The slope of each line corresponds to the estimated MR effect in different models. (A) Cigarettes per day; (B) Smoking initiation; (C) Past tobacco smoking; (D) Alcohol intake frequency; (E) Coffee intake. MR = Mendelian randomization, SNP = single nucleotide polymorphisms.

**Figure 5.**
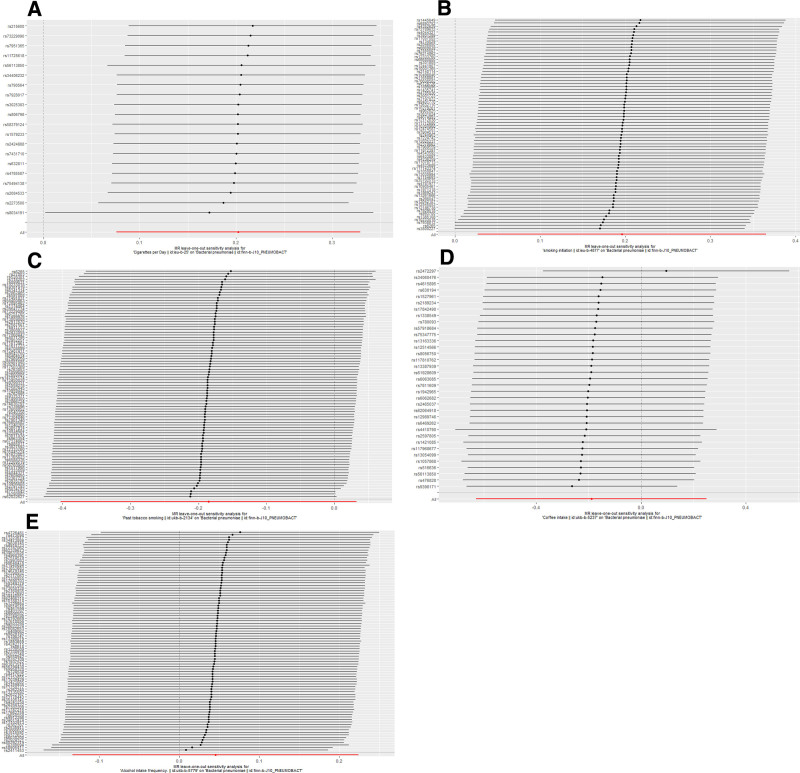
Leave-one-out of sensitivity tests. Calculate the MR results of the remaining IVs after removing the IVs one by one. (A) Cigarettes per day; (B) smoking initiation; (C) past tobacco smoking; (D) alcohol intake frequency; (E) coffee intake. IV = instrumental variant, MR = Mendelian randomization.

Detailed causal effect estimates for the association between exposure and outcome in the different models are presented in Figure 2 and Table 1. Forest plots and scatterplots for genetic prediction of the causal association between smoking, alcohol intake, and coffee intake with BP risk are presented in Figures 3 and 4. Details of the sensitivity analysis are presented in Figure 5 and Table 2. The above results suggest that there is a causal relationship between cigarettes per day, smoking initiation, and BP.

**Table 1:** Mendelian randomization estimates between lifestyle factors and BP.

Exposures*	Outcomes*	Method*	OR (95% CI)*	*P* value*
Cigarettes per day	BP	Inverse variance weighted	1.26 (1.08–1.39)	<.01
		Weighted median	1.27 (1.07–1.51)	.01
		MR Egger	1.27 (1.02–1.58)	.04
		Simple mode	1.27 (0.93–1.73)	.16
		Weighted mode	1.26 (1.06–1.49)	.02
Smoking initiation	BP	Inverse variance weighted	1.22 (1.03–1.44)	.02
		Weighted median	1.22 (0.95–1.56)	.11
		MR Egger	1.80 (0.77–4.20)	.18
		Simple mode	1.09 (0.60–1.97)	.78
		Weighted mode	1.20 (0.70–2.04)	.51
Past tobacco smoking	BP	Inverse variance weighted	0.83 (0.67–1.03)	.08
		Weighted median	0.96 (0.71–1.31)	.81
		MR Egger	0.35 (0.12–0.99)	.05
		Simple mode	1.22 (0.51–2.93)	.66
		Weighted mode	1.20 (0.56–2.57)	.64
Alcohol intake frequency	BP	Inverse variance weighted	0.83 (0.56–1.23)	.35
		Weighted median	0.88 (0.46–1.68)	.70
		MR Egger	0.40 (0.17–0.93)	.04
		Simple mode	1.07 (0.29–4.03)	.92
		Weighted mode	0.65 (0.33–1.28)	.22
Coffee intake	BP	Inverse variance weighted	1.05 (0.88–1.24)	.61
		Weighted median	1.04 (0.79–1.37)	.77
		MR Egger	1.31 (0.75–2.30)	.35
		Simple mode	1.01 (0.52–1.97)	.97
		Weighted mode	1.04 (0.64–1.70)	.88

BP = bacterial pneumonia, CI = confidence interval, MR = Mendelian randomization, OR = odds ratios.

* All figures were omitted to 2 decimal places.

**Table 2:** Heterogeneity and pleiotropy of 5 lifestyle factors in BP.

Exposures	Heterogeneity (*P* value)*	Pleiotropy (*P* value)*
Cigarettes per day	.94	.68
Smoking initiation	.49	.36
Past tobacco smoking	.29	.10
Alcohol intake frequency	.17	.41
Coffee intake	.31	.06

BP = bacterial pneumonia.

* All figures were omitted to 2 decimal places.

## 4. Discussion

Currently, the cost of treating BP in all age groups remains a huge burden on national health insurance. Therefore, it is valuable to explore controllable risk factors for BP and take measures to control risk factors. Smoking, alcohol intake, and coffee intake are the most common lifestyles associated with our health. Although increasing alcohol consumption, smoking, use of heated tobacco products, and other stimulants are common ways to cope with anxiety. However, they could have a serious negative impact on health.^[16]^ This 2-sample MR analysis found cigarettes per day and smoking initiation to be associated with an increased risk of BP. MR analysis failed to provide evidence of a causal relationship between past tobacco smoking, alcohol intake frequency, and coffee intake with BP.

Previous observational studies have suggested that smoking may influence the risk of respiratory infections, including coronavirus disease 2019.^[17]^ Possible mechanisms by which smoking increases the risk of respiratory infections include structural alterations in the respiratory tract and dysregulation of the cellular and humoral immune response, including peribronchial inflammation, decreased circulating immunoglobulin levels, and altered pathogen adhesion.^[18]^ Smoking also stimulates the release of catecholamines and corticosteroids, which may increase circulating CD8+ lymphocytes and inhibit host defenses against infection.^[5]^ Our genetics-based studies provide modest evidence for a causal association between cigarettes per day, smoking initiation, and BP risk. This result supports and extends the existing observational literature that smoking is a risk factor for respiratory infections^[19,20]^ and adds to the more recent MR literature that suggests a potential causal relationship between smoking and increased mortality from lung cancer, reduced lung function, and respiratory disease.^[21–23]^ Smokers are more inclined to engage in unhealthy lifestyles, including obesity and alcohol intake, which may have a negative impact on the prognosis of BP, although alcohol intake was not associated with BP in this MR analysis. Notably, our MR analysis did not find a causal relationship between past tobacco smoking and BP risk, suggesting that many smoking-related immune effects may disappear after smoking cessation and that smoking cessation programs may be beneficial in reducing BP risk.

Although the epidemiology of BP and excessive alcohol consumption is well established, the mechanisms by which alcohol induces the risk of BP are unclear. The pattern of alcohol abuse, known as alcohol use disorder (AUD), affects approximately 15 million people in the United States. AUD increases the risk of respiratory infections and acute respiratory distress syndrome by 2 to 4 times compared to otherwise healthy individuals. AUDs increase the risk of respiratory infections, which may be due to hyaluronic acid signaling through hyaluronan adhesions affected by alcohol use, altering inflammation and immune cell activity during BP.^[3]^ In addition, chronic alcohol abuse significantly impairs mucociliary clearance of bacterial pathogens from the upper respiratory tract^[24]^ and predisposes to BP by disrupting critical alveolar macrophage (AM) function.^[25]^ Currently, it has been widely accepted that the gut microbiota plays a crucial role in host immune responses to bacterial and viral respiratory infections.^[26–28]^ Alcohol increases the risk of pneumonia by disrupting gut microbiota balance. In addition, alcohol abuse increases the risk of microbial aspiration from the upper gastrointestinal tract. Chronic alcohol consumption also inhibited tissue recruitment of neutrophils during infection and inflammation,^[29]^ as well as the phagocytic capacity of AMs.^[30]^ Chronic alcohol consumption also decreases dendritic cell differentiation and effector function.^[31]^ Alcohol consumption also reduces the number of circulating lymphocytes and dysregulates Th1, Th2, and Th17 immune responses.^[32]^ Chronic ethanol consumption also disturbs pulmonary surfactant lipid homeostasis, and elevated concentrations of free fatty acids help to block essential AM functions, such as agonist-induced cytokine expression.^[25]^ The above studies showed that alcohol intake greatly increases the risk of pathogenic bacteria entering the lungs, thereby increasing the risk of infection. However, our MR analyses did not provide strong evidence for a causal relationship between alcohol intake and BP, which is also the same as the results of an MR analysis by Zhu et al.^[33]^ Therefore, the relationship between alcohol intake and BP may need to be explored in more studies.

The components contained in coffee have been reported to have a variety of health benefits. Caffeine contained in coffee has the effects of awakening, muscle strength, diuresis, and improving respiratory function, and its metabolite theophylline has the effects of dilating bronchi, stimulating respiratory centers, and anti-inflammatory.^[34]^ In addition, caffeine, chlorogenic acid, and trigonelline in coffee also have antibacterial activities.^[35–37]^ A large prospective cohort study in the United States showed that coffee intake was inversely associated with deaths from pneumonia, chronic respiratory disease, and influenza.^[38]^ Similarly, a case-control study in Japan showed that drinking more than 2 cups of coffee a day in older prevented the onset of pneumonia, and the study included 199 patients and 374 controls.^[39]^ Other cohort studies have also reported negative associations between coffee intake and death from respiratory diseases (such as pneumonia, influenza, and chronic obstructive pulmonary disease).^[40,41]^ These findings suggest that coffee may have a preventive effect on acute and chronic respiratory diseases. Up to now, our study is the first MR analysis of the association between coffee intake and BP. MR analysis found no causal relationship between coffee intake and BP, which provides a reference for future studies. This suggests that coffee intake may play a preventive role against BP through other pleiotropic factors, which needs to be explained by more studies.

The statistical efficacy of the IVW method is much greater than that of other MR methods, especially MR Egger method.^[42]^ In our study, the IVW method was used as the primary method for screening MR results. To ensure the robustness of our results, we also performed sensitivity analysis. Taken together, our findings support the hypothesis that cigarettes per day and smoking initiation can increase the risk of BP, and therefore strategies to reduce exposure to this factor deserve attention to reduce the risk of BP. Smoking cessation campaigns should be recommended in any age group to reduce the incidence of BP.

The present study has several strengths and limitations. Firstly, the majority of studies on smoking, alcohol intake, and coffee intake utilized self-reported consumption, which was easy to cause bias. However, This MR analysis examined summary statistics of several lifestyle factors from a large dataset. In our 2-sample MR analysis, genetic variation may examine the possible causative influence of exposures to BP without being biased or confounded by confounding or reserve causation. In addition, all studies included populations of predominantly European ancestry, which reduces the potential for bias related to population stratification but limits the applicability of our findings to other populations. Pleiotropy was ruled out, but alternative mechanisms for SNP and BP association may still exist. Of note, the age and sex of BP patients were not stratified in this study, so it was uncertain whether the effect of smoking on BP risk was influenced by age and sex. Another limitation was that the 2-sample MR design did not allow evaluation of the reverse causality of these exposures on BP. Finally, due to database limitations, we did not have access to specific data on the number of cigarettes smoked and coffee intake. In the future, we hope to make more efforts in this area.

## 5. Conclusion

The 2-sample MR analysis provided evidence for a causal relationship between genetically predicted cigarettes per day, smoking initiation, and an increased risk of BP. There were no associations of genetic liability to past tobacco smoking, alcohol intake frequency, and coffee intake with the risk of BP. Our results suggest that people who smoke cigarettes daily need special clinical attention to prevent the development of BP. Further studies are needed to test the biological mechanisms underlying this association.

## Acknowledgments

We sincerely thank the editor and all the reviewers for their efforts.

## Author contributions

**Formal analysis:** Zhendong He, Leting Zheng, Zhanrui Chen, Jing Wen, Fang Qin.

**Resources:** Zhendong He.

**Software:** Zhendong He.

**Writing – original draft:** Zhendong He.

**Data curation:** Leting Zheng, Zhanrui Chen, Jing Wen, Fang Qin.

**Conceptualization:** Hanyou Mo.

**Writing – review & editing:** Hanyou Mo.

## Supplementary Material


