# Supplementary material for: The causal association of smoking, alcohol intake, and coffee intake with the risk of bacterial pneumonia: A Mendelian randomization study
Source: Medicine (Baltimore). 2024 Dec 13;103(50):e40702. doi: 10.1097/MD.0000000000040702 (PMC11651526; doi:10.1097/MD.0000000000040702)
Supplement: Supplementary file 1 [file medi-103-e40702-s001.docx]

Supplementary Table 1. 93 SNPs associated with smoking initiation.

| SNP^*^ | Chromosome | Position | Effect  allele | Other allele | EAF | Beta | Se | P | Sample size | F |
| --- | --- | --- | --- | --- | --- | --- | --- | --- | --- | --- |
| rs3001723 | 1 | 44037685 | A | G | 0.3210 | 0.0335 | 0.0039 | 8.12E-18 | 632802 | 73.9 |
| rs7555507 | 1 | 73766037 | T | C | 0.4960 | -0.0241 | 0.0036 | 1.14E-11 | 632802 | 46.1 |
| rs6669839 | 1 | 50625979 | T | C | 0.2040 | 0.0260 | 0.0044 | 3.36E-09 | 632802 | 35.0 |
| rs12042107 | 1 | 91196176 | C | T | 0.5270 | -0.0223 | 0.0036 | 4.22E-10 | 632802 | 39.0 |
| rs2186122 | 1 | 66470206 | T | A | 0.5610 | 0.0261 | 0.0036 | 3.61E-13 | 632802 | 52.8 |
| rs301805 | 1 | 8481016 | G | T | 0.5590 | 0.0215 | 0.0036 | 2.80E-09 | 632802 | 35.3 |
| rs12025237 | 1 | 154205120 | C | A | 0.1240 | -0.0330 | 0.0053 | 6.52E-10 | 632802 | 38.2 |
| rs2050586 | 1 | 87905828 | C | G | 0.3550 | -0.0205 | 0.0037 | 3.00E-08 | 632802 | 30.7 |
| rs2046850 | 1 | 210304319 | T | C | 0.1870 | -0.0248 | 0.0045 | 3.03E-08 | 632802 | 30.7 |
| rs6728726 | 2 | 623976 | C | T | 0.8290 | 0.0354 | 0.0047 | 6.73E-14 | 632802 | 56.1 |
| rs78411160 | 2 | 58171220 | C | A | 0.6310 | 0.0205 | 0.0037 | 2.03E-08 | 632802 | 31.5 |
| rs6433897 | 2 | 182034448 | C | T | 0.7540 | 0.0224 | 0.0041 | 3.16E-08 | 632802 | 30.6 |
| rs266047 | 2 | 104088751 | A | G | 0.5290 | -0.0305 | 0.0037 | 3.36E-16 | 632802 | 66.6 |
| rs4674993 | 2 | 226332033 | G | A | 0.2070 | -0.0252 | 0.0044 | 1.32E-08 | 632802 | 32.3 |
| rs578584 | 2 | 45143175 | T | A | 0.6050 | 0.0287 | 0.0036 | 1.50E-15 | 632802 | 63.6 |
| rs35702515 | 2 | 137542847 | T | G | 0.1620 | 0.0252 | 0.0042 | 2.43E-09 | 632802 | 35.6 |
| rs13030994 | 2 | 146143090 | A | G | 0.4850 | 0.0361 | 0.0036 | 3.56E-24 | 632802 | 103.0 |
| rs12474587 | 2 | 162802993 | T | G | 0.4040 | 0.0276 | 0.0036 | 1.25E-14 | 632802 | 59.5 |
| rs2107300 | 2 | 200937901 | G | C | 0.8450 | -0.0272 | 0.0049 | 3.27E-08 | 632802 | 30.5 |
| rs7585579 | 2 | 60024857 | G | C | 0.5050 | 0.0224 | 0.0037 | 1.88E-09 | 632802 | 36.1 |
| rs1445649 | 2 | 155682556 | C | T | 0.5250 | 0.0240 | 0.0036 | 1.68E-11 | 632802 | 45.3 |
| rs6788098 | 3 | 85624131 | T | A | 0.6230 | -0.0313 | 0.0037 | 1.91E-17 | 632802 | 72.2 |
| rs12632110 | 3 | 50224225 | G | A | 0.6470 | -0.0234 | 0.0038 | 4.78E-10 | 632802 | 38.8 |
| rs11712680 | 3 | 75009019 | C | A | 0.1740 | -0.0270 | 0.0046 | 3.51E-09 | 632802 | 34.9 |
| rs1154693 | 3 | 117804154 | G | A | 0.8560 | 0.0326 | 0.0049 | 3.12E-11 | 632802 | 44.1 |
| rs66680800 | 3 | 85985324 | T | G | 0.3970 | -0.0203 | 0.0037 | 2.83E-08 | 632802 | 30.8 |
| rs1869243 | 3 | 5724536 | C | T | 0.4810 | 0.0197 | 0.0036 | 2.97E-08 | 632802 | 30.7 |
| rs9835772 | 3 | 85766025 | T | A | 0.2350 | 0.0240 | 0.0041 | 6.32E-09 | 632802 | 33.7 |
| rs962625 | 4 | 28473524 | G | A | 0.2400 | 0.0237 | 0.0040 | 4.37E-09 | 632802 | 34.5 |
| rs993700 | 4 | 67825894 | C | T | 0.7660 | -0.0259 | 0.0043 | 1.53E-09 | 632802 | 36.5 |
| rs13145728 | 4 | 140927812 | C | G | 0.3580 | -0.0233 | 0.0037 | 2.14E-10 | 632802 | 40.3 |
| rs10001365 | 4 | 147797214 | A | G | 0.4050 | -0.0250 | 0.0036 | 6.65E-12 | 632802 | 47.1 |
| rs1160685 | 4 | 94052854 | G | C | 0.4780 | 0.0208 | 0.0036 | 7.20E-09 | 632802 | 33.5 |
| rs6893752 | 5 | 60374912 | G | A | 0.7660 | -0.0241 | 0.0041 | 3.25E-09 | 632802 | 35.0 |
| rs12186738 | 5 | 103816655 | T | G | 0.1540 | -0.0333 | 0.0050 | 3.42E-11 | 632802 | 43.9 |
| rs1385108 | 5 | 154839646 | T | C | 0.2390 | 0.0247 | 0.0042 | 3.00E-09 | 632802 | 35.2 |
| rs4044321 | 5 | 166989513 | G | A | 0.6420 | -0.0278 | 0.0037 | 6.08E-14 | 632802 | 56.3 |
| rs4352629 | 5 | 87756821 | T | C | 0.4920 | -0.0275 | 0.0036 | 1.22E-14 | 632802 | 59.5 |
| rs72789632 | 5 | 106834363 | T | C | 0.1200 | -0.0329 | 0.0053 | 5.02E-10 | 632802 | 38.7 |
| rs9401770 | 6 | 98748008 | A | G | 0.2730 | 0.0277 | 0.0040 | 3.47E-12 | 632802 | 48.4 |
| rs222449 | 6 | 52916062 | T | A | 0.7930 | -0.0253 | 0.0044 | 1.08E-08 | 632802 | 32.7 |
| rs3800227 | 6 | 108994161 | G | A | 0.7010 | 0.0228 | 0.0041 | 1.93E-08 | 632802 | 31.6 |
| rs10498846 | 6 | 67405337 | T | C | 0.4730 | 0.0206 | 0.0036 | 6.62E-09 | 632802 | 33.6 |
| rs240963 | 6 | 111644332 | C | T | 0.8360 | -0.0410 | 0.0048 | 2.16E-17 | 632802 | 72.0 |
| rs12333760 | 7 | 99185406 | C | T | 0.2040 | -0.0290 | 0.0048 | 1.44E-09 | 632802 | 36.6 |
| rs10233018 | 7 | 117523709 | G | A | 0.5030 | 0.0271 | 0.0036 | 2.75E-14 | 632802 | 57.9 |
| rs10279261 | 7 | 133589846 | A | G | 0.6190 | -0.0214 | 0.0037 | 5.00E-09 | 632802 | 34.2 |
| rs10260968 | 7 | 1889773 | A | G | 0.5970 | -0.0203 | 0.0036 | 1.75E-08 | 632802 | 31.7 |
| rs12112638 | 7 | 69735251 | G | A | 0.2750 | -0.0245 | 0.0040 | 1.34E-09 | 632802 | 36.8 |
| rs4236259 | 7 | 1708080 | G | T | 0.4990 | -0.0248 | 0.0036 | 3.35E-12 | 632802 | 48.5 |
| rs2140114 | 7 | 3407568 | T | C | 0.5180 | -0.0233 | 0.0037 | 4.70E-10 | 632802 | 38.8 |
| rs3801289 | 7 | 96638267 | C | A | 0.3510 | -0.0221 | 0.0037 | 3.74E-09 | 632802 | 34.8 |
| rs1565735 | 8 | 27426077 | A | T | 0.2120 | -0.0376 | 0.0045 | 3.42E-17 | 632802 | 71.1 |
| rs1899896 | 8 | 93201036 | T | C | 0.2860 | 0.0264 | 0.0039 | 1.04E-11 | 632802 | 46.3 |
| rs13261666 | 8 | 59814666 | T | G | 0.5220 | -0.0269 | 0.0036 | 3.90E-14 | 632802 | 57.2 |
| rs12545053 | 8 | 65073605 | G | A | 0.3970 | 0.0203 | 0.0036 | 2.43E-08 | 632802 | 31.1 |
| rs2631024 | 8 | 91995577 | G | A | 0.7370 | -0.0230 | 0.0040 | 1.18E-08 | 632802 | 32.5 |
| rs4543592 | 9 | 3014254 | C | T | 0.4680 | 0.0219 | 0.0036 | 7.46E-10 | 632802 | 37.9 |
| rs2378662 | 9 | 86707289 | A | G | 0.5560 | 0.0209 | 0.0036 | 4.16E-09 | 632802 | 34.5 |
| rs10114490 | 9 | 11070165 | A | G | 0.1980 | -0.0255 | 0.0045 | 1.81E-08 | 632802 | 31.7 |
| rs10905461 | 10 | 8803551 | C | T | 0.7180 | -0.0240 | 0.0041 | 7.35E-09 | 632802 | 33.4 |
| rs7921378 | 10 | 63674885 | C | G | 0.4630 | -0.0255 | 0.0036 | 8.26E-13 | 632802 | 51.2 |
| rs12356821 | 10 | 104563808 | C | G | 0.1400 | 0.0394 | 0.0050 | 6.27E-15 | 632802 | 60.8 |
| rs10159545 | 10 | 21766969 | G | C | 0.3750 | 0.0263 | 0.0037 | 1.84E-12 | 632802 | 49.6 |
| rs9423279 | 10 | 125680419 | G | C | 0.6410 | -0.0205 | 0.0037 | 3.21E-08 | 632802 | 30.6 |
| rs7938812 | 11 | 112911004 | G | T | 0.4240 | 0.0438 | 0.0036 | 2.71E-33 | 632802 | 145.0 |
| rs6265 | 11 | 27679916 | T | C | 0.2030 | -0.0318 | 0.0046 | 3.77E-12 | 632802 | 48.2 |
| rs7929518 | 11 | 85980958 | G | A | 0.7650 | 0.0242 | 0.0043 | 1.56E-08 | 632802 | 32.0 |
| rs4523689 | 11 | 7950797 | G | A | 0.4080 | -0.0206 | 0.0036 | 1.55E-08 | 632802 | 32.0 |
| rs11057005 | 12 | 16748721 | G | A | 0.4300 | -0.0209 | 0.0036 | 4.85E-09 | 632802 | 34.2 |
| rs4759228 | 12 | 56508409 | C | G | 0.2700 | -0.0217 | 0.0039 | 3.58E-08 | 632802 | 30.4 |
| rs7969559 | 12 | 69655167 | G | A | 0.6880 | -0.0244 | 0.0040 | 7.31E-10 | 632802 | 37.9 |
| rs1971318 | 12 | 121389500 | T | C | 0.1410 | 0.0285 | 0.0049 | 7.06E-09 | 632802 | 33.5 |
| rs7322872 | 13 | 100548329 | T | C | 0.7820 | -0.0256 | 0.0043 | 3.58E-09 | 632802 | 34.8 |
| rs3904512 | 13 | 38357471 | A | G | 0.4290 | -0.0212 | 0.0036 | 3.23E-09 | 632802 | 35.0 |
| rs9540729 | 13 | 66947124 | T | A | 0.5010 | -0.0196 | 0.0036 | 3.82E-08 | 632802 | 30.2 |
| rs76214862 | 14 | 29500130 | C | A | 0.2020 | -0.0250 | 0.0045 | 3.99E-08 | 632802 | 30.2 |
| rs12441907 | 15 | 83922387 | A | C | 0.1860 | -0.0292 | 0.0045 | 1.06E-10 | 632802 | 41.7 |
| rs1435741 | 15 | 47935843 | A | G | 0.4250 | 0.0294 | 0.0036 | 2.64E-16 | 632802 | 67.1 |
| rs4785836 | 16 | 65604652 | C | T | 0.3980 | -0.0205 | 0.0037 | 2.26E-08 | 632802 | 31.3 |
| rs7197072 | 16 | 717085 | T | C | 0.2380 | -0.0248 | 0.0042 | 2.77E-09 | 632802 | 35.3 |
| rs1050847 | 16 | 87443734 | T | C | 0.5050 | -0.0216 | 0.0036 | 1.67E-09 | 632802 | 36.3 |
| rs4781977 | 16 | 17572674 | C | T | 0.2050 | -0.0239 | 0.0044 | 4.54E-08 | 632802 | 29.9 |
| rs11078713 | 17 | 7795972 | G | A | 0.4540 | -0.0202 | 0.0036 | 2.23E-08 | 632802 | 31.3 |
| rs7224742 | 17 | 30657058 | T | C | 0.5950 | -0.0207 | 0.0037 | 1.43E-08 | 632802 | 32.1 |
| rs11658881 | 17 | 2072949 | G | A | 0.4180 | 0.0201 | 0.0036 | 2.43E-08 | 632802 | 31.1 |
| rs6508144 | 18 | 50026142 | G | C | 0.5630 | -0.0207 | 0.0036 | 7.97E-09 | 632802 | 33.3 |
| rs11872397 | 18 | 72535282 | A | G | 0.2520 | -0.0248 | 0.0041 | 1.43E-09 | 632802 | 36.6 |
| rs72896886 | 18 | 42632652 | C | G | 0.1440 | -0.0269 | 0.0048 | 2.75E-08 | 632802 | 30.9 |
| rs76608582 | 19 | 4474725 | A | C | 0.0389 | -0.0496 | 0.0083 | 1.94E-09 | 632802 | 36.0 |
| rs1555445 | 20 | 31175258 | T | A | 0.3370 | 0.0226 | 0.0038 | 3.65E-09 | 632802 | 34.8 |
| rs117143374 | 21 | 40555561 | C | T | 0.1200 | 0.0293 | 0.0053 | 2.76E-08 | 632802 | 30.9 |
| rs134529 | 22 | 28781758 | C | T | 0.3490 | -0.0200 | 0.0037 | 4.85E-08 | 632802 | 29.8 |

* SNP, single nucleotide polymorphisms.

Supplementary Table 2. 23 SNPs associated with cigarettes per day.

| SNP^*^ | Chromosome | Position | Effect allele | Other allele | EAF | Beta | Se | P | Sample size | F |
| --- | --- | --- | --- | --- | --- | --- | --- | --- | --- | --- |
| rs2072659 | 1 | 154548521 | G | C | 0.1050 | -0.0653 | 0.0092 | 1.71E-12 | 225752 | 49.8 |
| rs2084533 | 3 | 16872929 | T | C | 0.3190 | 0.0336 | 0.0059 | 1.22E-08 | 260706 | 32.5 |
| rs7431710 | 3 | 48935583 | A | G | 0.6440 | -0.0350 | 0.0058 | 1.82E-09 | 261833 | 36.2 |
| rs787362 | 4 | 67904931 | A | T | 0.4520 | 0.0305 | 0.0056 | 4.50E-08 | 262450 | 29.9 |
| rs11725618 | 4 | 67053769 | C | T | 0.2870 | 0.0361 | 0.0062 | 4.67E-09 | 250822 | 34.3 |
| rs806798 | 6 | 26214473 | C | T | 0.5430 | -0.0309 | 0.0055 | 2.48E-08 | 257120 | 31.1 |
| rs215600 | 7 | 32333642 | A | G | 0.6400 | -0.0493 | 0.0058 | 1.10E-17 | 262746 | 73.3 |
| rs58379124 | 8 | 42579203 | C | T | 0.7480 | 0.0669 | 0.0065 | 9.00E-25 | 258370 | 106.0 |
| rs790564 | 8 | 64604218 | C | A | 0.7190 | -0.0409 | 0.0062 | 3.97E-11 | 261674 | 43.6 |
| rs73229090 | 8 | 27442127 | A | C | 0.1130 | 0.0555 | 0.0088 | 2.44E-10 | 255628 | 40.1 |
| rs3025383 | 9 | 136502369 | C | T | 0.1800 | -0.0578 | 0.0070 | 2.22E-16 | 256134 | 67.4 |
| rs75494138 | 11 | 46465361 | T | C | 0.0618 | 0.0599 | 0.0106 | 1.45E-08 | 258399 | 32.1 |
| rs7928017 | 11 | 113448762 | A | C | 0.4130 | -0.0329 | 0.0056 | 3.14E-09 | 258763 | 35.1 |
| rs7951365 | 11 | 16377044 | C | T | 0.3060 | 0.0390 | 0.0060 | 6.63E-11 | 258295 | 42.6 |
| rs632811 | 15 | 59155050 | G | A | 0.3510 | -0.0367 | 0.0064 | 1.03E-08 | 214251 | 32.8 |
| rs8034191 | 15 | 78806023 | C | T | 0.3280 | 0.1826 | 0.0059 | 1.00E-200 | 255729 | 961.0 |
| rs1579233 | 16 | 52074530 | G | A | 0.5710 | -0.0318 | 0.0056 | 1.07E-08 | 259482 | 32.7 |
| rs4785587 | 16 | 89772619 | A | G | 0.5110 | -0.0336 | 0.0055 | 1.27E-09 | 254249 | 36.9 |
| rs895330 | 19 | 4060707 | G | C | 0.2060 | -0.0390 | 0.0070 | 2.68E-08 | 250059 | 30.9 |
| rs34406232 | 19 | 41305530 | A | C | 0.0259 | -0.1470 | 0.0167 | 1.33E-18 | 251880 | 77.5 |
| rs56113850 | 19 | 41353107 | C | T | 0.5680 | 0.1072 | 0.0056 | 1.10E-81 | 243952 | 366.0 |
| rs2273500 | 20 | 61986949 | C | T | 0.1590 | 0.0681 | 0.0078 | 2.47E-18 | 252983 | 76.3 |
| rs2424888 | 20 | 31047533 | A | G | 0.4050 | 0.0335 | 0.0056 | 2.76E-09 | 257430 | 35.3 |

* SNP, single nucleotide polymorphisms.

Supplementary Table 3. 101 SNPs associated with past tobacco smoking.

| SNP | Chromosome | Position | Effect allele | Other allele | EAF | Beta | Se | P | Sample size | F |
| --- | --- | --- | --- | --- | --- | --- | --- | --- | --- | --- |
| rs2186122 | 1 | 66470206 | T | A | 0.5594 | -0.0157 | 0.0027 | 9.00E-09 | 424960 | 33.1 |
| rs17503369 | 1 | 73847108 | C | T | 0.1822 | 0.0216 | 0.0035 | 6.60E-10 | 424960 | 38.1 |
| rs77068442 | 1 | 158845316 | G | A | 0.1072 | -0.0239 | 0.0044 | 4.10E-08 | 424960 | 30.1 |
| rs10914684 | 1 | 33795572 | A | G | 0.3255 | 0.0166 | 0.0029 | 7.80E-09 | 424960 | 33.3 |
| rs2367724 | 1 | 44107428 | T | C | 0.6736 | 0.0160 | 0.0029 | 2.40E-08 | 424960 | 31.1 |
| rs11165623 | 1 | 96893000 | A | G | 0.5041 | -0.0158 | 0.0027 | 4.40E-09 | 424960 | 34.4 |
| rs35761479 | 1 | 154154194 | A | G | 0.1208 | 0.0230 | 0.0041 | 2.80E-08 | 424960 | 30.8 |
| rs6588376 | 1 | 50602495 | A | G | 0.2083 | -0.0188 | 0.0033 | 1.40E-08 | 424960 | 32.2 |
| rs3935790 | 1 | 208725458 | A | G | 0.4161 | -0.0155 | 0.0027 | 1.60E-08 | 424960 | 31.9 |
| rs1040070 | 1 | 74977870 | C | G | 0.5688 | 0.0163 | 0.0027 | 2.50E-09 | 424960 | 35.6 |
| rs147052174 | 1 | 179783167 | T | G | 0.0186 | -0.0604 | 0.0100 | 1.40E-09 | 424960 | 36.7 |
| rs74676797 | 2 | 633063 | A | G | 0.8081 | -0.0233 | 0.0035 | 3.60E-11 | 424960 | 43.8 |
| rs528301 | 2 | 45154908 | A | G | 0.5543 | -0.0203 | 0.0027 | 5.60E-14 | 424960 | 56.5 |
| rs7582445 | 2 | 60495874 | C | A | 0.5878 | -0.0172 | 0.0027 | 3.20E-10 | 424960 | 39.6 |
| rs7609050 | 2 | 156021862 | C | A | 0.5256 | -0.0158 | 0.0027 | 5.00E-09 | 424960 | 34.2 |
| rs11693702 | 2 | 162802184 | A | T | 0.4631 | -0.0172 | 0.0027 | 2.20E-10 | 424960 | 40.3 |
| rs67174662 | 2 | 59295476 | G | A | 0.3753 | 0.0167 | 0.0028 | 2.30E-09 | 424960 | 35.7 |
| rs1492546 | 2 | 81013736 | G | C | 0.5529 | -0.0155 | 0.0027 | 1.20E-08 | 424960 | 32.5 |
| rs7596680 | 2 | 146114071 | G | C | 0.5354 | 0.0269 | 0.0027 | 2.00E-23 | 424960 | 99.5 |
| rs7600005 | 2 | 48212055 | A | G | 0.3734 | 0.0156 | 0.0028 | 2.60E-08 | 424960 | 31.0 |
| rs290882 | 2 | 118262987 | C | T | 0.2452 | 0.0175 | 0.0031 | 2.30E-08 | 424960 | 31.2 |
| rs13009008 | 2 | 174043233 | G | A | 0.6727 | 0.0158 | 0.0029 | 3.90E-08 | 424960 | 30.2 |
| rs6751705 | 2 | 104113702 | G | T | 0.5121 | -0.0225 | 0.0027 | 7.10E-17 | 424960 | 69.6 |
| rs3811038 | 2 | 113240183 | C | T | 0.2760 | -0.0171 | 0.0030 | 1.80E-08 | 424960 | 31.7 |
| rs67336646 | 3 | 85524474 | A | T | 0.6269 | 0.0236 | 0.0028 | 1.80E-17 | 424960 | 72.3 |
| rs9835772 | 3 | 85766025 | T | A | 0.2436 | -0.0183 | 0.0031 | 5.70E-09 | 424960 | 33.9 |
| rs12487411 | 3 | 34422170 | A | G | 0.4708 | 0.0168 | 0.0027 | 5.00E-10 | 424960 | 38.7 |
| rs963354 | 3 | 157393770 | A | C | 0.6734 | -0.0176 | 0.0029 | 8.70E-10 | 424960 | 37.6 |
| rs1499976 | 3 | 117801330 | C | T | 0.8527 | -0.0314 | 0.0038 | 1.90E-16 | 424960 | 67.7 |
| rs56760958 | 3 | 85983138 | T | C | 0.3903 | 0.0173 | 0.0028 | 3.80E-10 | 424960 | 39.2 |
| rs899631 | 4 | 57749363 | T | G | 0.3912 | 0.0177 | 0.0028 | 1.80E-10 | 424960 | 40.7 |
| rs77304846 | 4 | 70495353 | C | T | 0.1830 | 0.0209 | 0.0035 | 2.10E-09 | 424960 | 35.9 |
| rs58400863 | 4 | 31184484 | A | G | 0.3416 | 0.0176 | 0.0029 | 6.50E-10 | 424960 | 38.2 |
| rs3827592 | 4 | 147948150 | A | G | 0.3510 | 0.0177 | 0.0028 | 3.80E-10 | 424960 | 39.2 |
| rs6828849 | 4 | 173077123 | T | A | 0.4188 | 0.0179 | 0.0027 | 5.70E-11 | 424960 | 42.9 |
| rs1559278 | 5 | 50794221 | C | T | 0.3612 | 0.0155 | 0.0028 | 3.40E-08 | 424960 | 30.5 |
| rs7733542 | 5 | 106410664 | G | A | 0.6294 | -0.0157 | 0.0028 | 2.50E-08 | 424960 | 31.0 |
| rs1017998 | 5 | 79263211 | G | A | 0.6204 | -0.0152 | 0.0028 | 4.40E-08 | 424960 | 30.0 |
| rs27003 | 5 | 94202167 | C | T | 0.6951 | -0.0164 | 0.0029 | 2.20E-08 | 424960 | 31.3 |
| rs10474278 | 5 | 87841490 | G | A | 0.7468 | -0.0185 | 0.0031 | 3.00E-09 | 424960 | 35.2 |
| rs4044321 | 5 | 166989513 | G | A | 0.6419 | 0.0184 | 0.0028 | 6.90E-11 | 424960 | 42.6 |
| rs1611723 | 6 | 29830505 | G | A | 0.4051 | 0.0158 | 0.0027 | 7.30E-09 | 424960 | 33.4 |
| rs4708899 | 6 | 157751114 | G | A | 0.5756 | 0.0159 | 0.0027 | 6.20E-09 | 424960 | 33.8 |
| rs9381919 | 6 | 50931059 | T | G | 0.1036 | 0.0271 | 0.0044 | 9.60E-10 | 424960 | 37.4 |
| rs2797793 | 6 | 37477262 | C | T | 0.6039 | 0.0153 | 0.0028 | 3.00E-08 | 424960 | 30.7 |
| rs12209519 | 6 | 67549140 | G | A | 0.4076 | -0.0159 | 0.0028 | 8.00E-09 | 424960 | 33.3 |
| rs9375371 | 6 | 98751680 | A | G | 0.2694 | -0.0199 | 0.0030 | 6.30E-11 | 424960 | 42.7 |
| rs118202 | 6 | 111658371 | T | G | 0.8174 | 0.0324 | 0.0035 | 1.20E-20 | 424960 | 86.8 |
| rs885011 | 7 | 3484778 | C | T | 0.5013 | 0.0203 | 0.0027 | 5.40E-14 | 424960 | 56.6 |
| rs1499300 | 7 | 132311000 | C | A | 0.1579 | 0.0218 | 0.0037 | 3.60E-09 | 424960 | 34.8 |
| rs1899689 | 7 | 121964349 | T | C | 0.3891 | -0.0154 | 0.0028 | 2.30E-08 | 424960 | 31.2 |
| rs12333760 | 7 | 99185406 | C | T | 0.1655 | 0.0214 | 0.0036 | 3.90E-09 | 424960 | 34.7 |
| rs1174864 | 7 | 53127559 | A | G | 0.5499 | -0.0154 | 0.0027 | 1.50E-08 | 424960 | 32.0 |
| rs6464024 | 7 | 1688369 | T | C | 0.4274 | 0.0176 | 0.0027 | 1.10E-10 | 424960 | 41.6 |
| rs10233018 | 7 | 117523709 | G | A | 0.5030 | -0.0151 | 0.0027 | 1.90E-08 | 424960 | 31.6 |
| rs73229090 | 8 | 27442127 | A | C | 0.1179 | 0.0289 | 0.0042 | 8.40E-12 | 424960 | 46.7 |
| rs3857914 | 8 | 93184065 | C | T | 0.3021 | -0.0202 | 0.0030 | 9.60E-12 | 424960 | 46.4 |
| rs2433055 | 8 | 133769805 | G | T | 0.4477 | 0.0148 | 0.0027 | 4.60E-08 | 424960 | 29.9 |
| rs2952251 | 8 | 10143164 | G | A | 0.7399 | -0.0175 | 0.0031 | 1.20E-08 | 424960 | 32.4 |
| rs10956808 | 8 | 92775372 | G | T | 0.4221 | 0.0189 | 0.0027 | 4.90E-12 | 424960 | 47.7 |
| rs10959442 | 9 | 10993737 | G | T | 0.4664 | -0.0172 | 0.0027 | 1.90E-10 | 424960 | 40.6 |
| rs1246265 | 9 | 86761745 | C | T | 0.6952 | -0.0209 | 0.0029 | 1.00E-12 | 424960 | 50.8 |
| rs28647734 | 9 | 137977033 | A | G | 0.2100 | -0.0192 | 0.0033 | 8.30E-09 | 424960 | 33.2 |
| rs9299331 | 9 | 102146915 | C | T | 0.5261 | -0.0175 | 0.0027 | 9.10E-11 | 424960 | 42.0 |
| rs9423279 | 10 | 125680419 | G | C | 0.6571 | 0.0164 | 0.0029 | 1.30E-08 | 424960 | 32.3 |
| rs7901348 | 10 | 63679281 | G | T | 0.5523 | 0.0193 | 0.0027 | 1.60E-12 | 424960 | 50.0 |
| rs12244388 | 10 | 104640052 | A | G | 0.3381 | -0.0255 | 0.0028 | 3.30E-19 | 424960 | 80.2 |
| rs911773 | 10 | 123964980 | C | A | 0.4895 | 0.0153 | 0.0027 | 1.50E-08 | 424960 | 32.1 |
| rs3808937 | 10 | 104230012 | T | C | 0.2078 | -0.0205 | 0.0033 | 6.20E-10 | 424960 | 38.2 |
| rs2862465 | 11 | 42379492 | A | G | 0.4092 | 0.0158 | 0.0027 | 7.30E-09 | 424960 | 33.5 |
| rs2155290 | 11 | 112851068 | G | C | 0.3836 | -0.0395 | 0.0028 | 4.60E-46 | 424960 | 203.0 |
| rs540356 | 11 | 132203816 | A | C | 0.4136 | -0.0184 | 0.0028 | 2.40E-11 | 424960 | 44.6 |
| rs6265 | 11 | 27679916 | T | C | 0.1890 | 0.0261 | 0.0034 | 3.00E-14 | 424960 | 57.8 |
| rs71491831 | 11 | 124605783 | A | G | 0.0757 | 0.0306 | 0.0051 | 2.00E-09 | 424960 | 35.9 |
| rs7969559 | 12 | 69655167 | G | A | 0.7205 | 0.0173 | 0.0030 | 7.50E-09 | 424960 | 33.4 |
| rs597808 | 12 | 111973358 | G | A | 0.5158 | 0.0221 | 0.0027 | 2.60E-16 | 424960 | 67.1 |
| rs11613961 | 12 | 133474880 | C | T | 0.0868 | -0.0294 | 0.0048 | 9.30E-10 | 424960 | 37.5 |
| rs1109480 | 12 | 121083279 | A | G | 0.3891 | 0.0163 | 0.0028 | 4.50E-09 | 424960 | 34.4 |
| rs56081685 | 13 | 59454140 | G | T | 0.3137 | 0.0165 | 0.0029 | 1.40E-08 | 424960 | 32.1 |
| rs837335 | 13 | 101180197 | C | G | 0.4903 | -0.0151 | 0.0027 | 2.10E-08 | 424960 | 31.4 |
| rs7333559 | 13 | 100546450 | A | G | 0.7887 | 0.0200 | 0.0033 | 1.80E-09 | 424960 | 36.2 |
| rs9542750 | 13 | 72393720 | C | T | 0.5857 | 0.0152 | 0.0028 | 3.60E-08 | 424960 | 30.3 |
| rs12895462 | 14 | 77615441 | C | T | 0.1914 | 0.0200 | 0.0034 | 6.30E-09 | 424960 | 33.7 |
| rs62022627 | 15 | 89904546 | G | A | 0.4006 | 0.0190 | 0.0028 | 5.90E-12 | 424960 | 47.4 |
| rs8034783 | 15 | 47763726 | T | C | 0.1010 | -0.0282 | 0.0045 | 3.30E-10 | 424960 | 39.5 |
| rs2289791 | 15 | 67476952 | T | G | 0.2473 | 0.0191 | 0.0031 | 1.30E-09 | 424960 | 36.8 |
| rs763053 | 16 | 735921 | C | T | 0.2261 | 0.0195 | 0.0032 | 1.70E-09 | 424960 | 36.3 |
| rs2866724 | 16 | 13760152 | G | A | 0.2663 | -0.0188 | 0.0031 | 7.30E-10 | 424960 | 37.9 |
| rs77878475 | 16 | 18058548 | A | T | 0.0843 | 0.0331 | 0.0050 | 4.80E-11 | 424960 | 43.3 |
| rs2917670 | 16 | 69758963 | C | T | 0.6102 | 0.0168 | 0.0028 | 1.30E-09 | 424960 | 36.8 |
| rs35445224 | 16 | 73084276 | C | T | 0.1810 | -0.0206 | 0.0036 | 8.10E-09 | 424960 | 33.3 |
| rs12450028 | 17 | 2207425 | T | C | 0.3451 | -0.0166 | 0.0028 | 4.60E-09 | 424960 | 34.4 |
| rs7216173 | 17 | 51891405 | T | A | 0.7821 | 0.0208 | 0.0033 | 3.40E-10 | 424960 | 39.4 |
| rs8071295 | 17 | 50131994 | A | C | 0.1573 | 0.0225 | 0.0037 | 1.40E-09 | 424960 | 36.7 |
| rs2587507 | 17 | 77790135 | C | T | 0.5057 | 0.0156 | 0.0027 | 5.90E-09 | 424960 | 33.9 |
| rs1623003 | 18 | 21165163 | T | C | 0.6640 | -0.0185 | 0.0029 | 1.20E-10 | 424960 | 41.5 |
| rs12608052 | 18 | 49803160 | T | C | 0.5189 | 0.0161 | 0.0027 | 2.40E-09 | 424960 | 35.6 |
| rs76608582 | 19 | 4474725 | A | C | 0.0473 | 0.0430 | 0.0067 | 1.10E-10 | 424960 | 41.5 |
| rs6141314 | 20 | 31093514 | A | G | 0.2416 | -0.0192 | 0.0032 | 1.50E-09 | 424960 | 36.6 |
| rs762995 | 22 | 42672124 | G | A | 0.5351 | 0.0149 | 0.0027 | 3.70E-08 | 424960 | 30.3 |
| rs139896 | 22 | 38397797 | C | T | 0.6478 | -0.0162 | 0.0028 | 9.00E-09 | 424960 | 33.1 |

* SNP, single nucleotide polymorphisms.

Supplementary Table 4. 99 SNPs associated with alcohol intake frequency.

| SNP | Chromosome | Position | Effect allele | Other allele | EAF | Beta | | Se | P | Sample size | | F | |
| --- | --- | --- | --- | --- | --- | --- | --- | --- | --- | --- | --- | --- | --- |
| rs780569 | 1 | 4569436 | A | T | 0.7088 | | 0.0198 | 0.0034 | 4.00E-09 | | 462346 | | 34.6 |
| rs4503294 | 1 | 940096 | T | C | 0.5653 | | 0.0181 | 0.0031 | 3.40E-09 | | 462346 | | 34.9 |
| rs28787109 | 1 | 51218695 | A | G | 0.4042 | | 0.0178 | 0.0031 | 7.70E-09 | | 462346 | | 33.3 |
| rs2244598 | 1 | 216681000 | C | T | 0.6051 | | -0.0184 | 0.0031 | 3.80E-09 | | 462346 | | 34.7 |
| rs4417025 | 1 | 35363679 | A | G | 0.3612 | | -0.0188 | 0.0032 | 2.70E-09 | | 462346 | | 35.4 |
| rs7514579 | 1 | 94051350 | C | A | 0.2325 | | 0.0197 | 0.0036 | 4.60E-08 | | 462346 | | 29.9 |
| rs2717063 | 2 | 58110969 | A | C | 0.5857 | | -0.0204 | 0.0031 | 4.00E-11 | | 462346 | | 43.6 |
| rs6727281 | 2 | 65558588 | T | C | 0.1840 | | -0.0243 | 0.0039 | 5.50E-10 | | 462346 | | 38.5 |
| rs780094 | 2 | 27741237 | C | T | 0.6152 | | -0.0510 | 0.0031 | 1.30E-60 | | 462346 | | 269.7 |
| rs13390019 | 2 | 97797680 | C | T | 0.1340 | | 0.0296 | 0.0045 | 4.30E-11 | | 462346 | | 43.5 |
| rs10188314 | 2 | 215402926 | T | C | 0.4709 | | -0.0198 | 0.0030 | 7.20E-11 | | 462346 | | 42.5 |
| rs4241258 | 2 | 74226102 | T | C | 0.1376 | | 0.0251 | 0.0044 | 1.30E-08 | | 462346 | | 32.4 |
| rs72769229 | 2 | 2220795 | T | A | 0.1549 | | -0.0231 | 0.0042 | 3.40E-08 | | 462346 | | 30.5 |
| rs17662759 | 2 | 193989223 | C | T | 0.0891 | | 0.0301 | 0.0055 | 3.40E-08 | | 462346 | | 30.5 |
| rs1991083 | 2 | 23887437 | T | C | 0.6799 | | -0.0224 | 0.0033 | 6.30E-12 | | 462346 | | 47.2 |
| rs473098 | 2 | 45139779 | T | C | 0.5577 | | -0.0217 | 0.0030 | 9.10E-13 | | 462346 | | 51.0 |
| rs9829192 | 3 | 38569463 | T | G | 0.4351 | | 0.0169 | 0.0031 | 2.80E-08 | | 462346 | | 30.8 |
| rs76082653 | 3 | 49029468 | T | C | 0.0543 | | 0.0464 | 0.0067 | 3.80E-12 | | 462346 | | 48.2 |
| rs262240 | 3 | 68408109 | T | C | 0.4686 | | -0.0172 | 0.0030 | 1.40E-08 | | 462346 | | 32.1 |
| rs9814516 | 3 | 85407980 | T | G | 0.2374 | | -0.0251 | 0.0036 | 1.60E-12 | | 462346 | | 49.9 |
| rs7610856 | 3 | 71579022 | A | C | 0.4291 | | -0.0239 | 0.0031 | 7.70E-15 | | 462346 | | 60.4 |
| rs1515591 | 3 | 174213976 | G | T | 0.3832 | | 0.0182 | 0.0031 | 4.90E-09 | | 462346 | | 34.2 |
| rs1228589 | 3 | 131634826 | A | G | 0.2461 | | 0.0211 | 0.0035 | 2.30E-09 | | 462346 | | 35.7 |
| rs28622224 | 4 | 55088093 | T | C | 0.2804 | | -0.0186 | 0.0034 | 3.20E-08 | | 462346 | | 30.6 |
| rs13135092 | 4 | 103198082 | G | A | 0.0835 | | 0.0438 | 0.0055 | 1.60E-15 | | 462346 | | 63.5 |
| rs11940694 | 4 | 39414993 | G | A | 0.6042 | | -0.0437 | 0.0031 | 1.00E-44 | | 462346 | | 196.8 |
| rs362307 | 4 | 3241845 | T | C | 0.0746 | | 0.0433 | 0.0058 | 8.40E-14 | | 462346 | | 55.7 |
| rs1229984 | 4 | 100239319 | C | T | 0.9728 | | -0.2617 | 0.0092 | 1.40E-178 | | 462346 | | 811.9 |
| rs13102973 | 4 | 135900688 | C | T | 0.6188 | | -0.0194 | 0.0031 | 4.90E-10 | | 462346 | | 38.7 |
| rs62339673 | 4 | 184828533 | A | C | 0.6267 | | 0.0183 | 0.0032 | 6.60E-09 | | 462346 | | 33.6 |
| rs34811474 | 4 | 25408838 | A | G | 0.2307 | | -0.0202 | 0.0036 | 1.90E-08 | | 462346 | | 31.5 |
| rs2159935 | 4 | 55521017 | A | G | 0.4904 | | -0.0186 | 0.0030 | 8.30E-10 | | 462346 | | 37.7 |
| rs62305780 | 4 | 100290815 | G | C | 0.1023 | | -0.0485 | 0.0051 | 9.90E-22 | | 462346 | | 91.7 |
| rs13178443 | 5 | 145615275 | T | C | 0.2763 | | -0.0187 | 0.0034 | 3.80E-08 | | 462346 | | 30.3 |
| rs11750777 | 5 | 166830787 | A | G | 0.2095 | | -0.0205 | 0.0037 | 3.80E-08 | | 462346 | | 30.2 |
| rs4916723 | 5 | 87854395 | C | A | 0.4206 | | 0.0239 | 0.0031 | 1.10E-14 | | 462346 | | 59.7 |
| rs461599 | 5 | 144136931 | C | A | 0.4623 | | -0.0192 | 0.0030 | 2.70E-10 | | 462346 | | 39.8 |
| rs56194430 | 5 | 67824690 | T | C | 0.1693 | | 0.0225 | 0.0041 | 3.10E-08 | | 462346 | | 30.6 |
| rs9403297 | 6 | 141705482 | A | G | 0.3730 | | 0.0188 | 0.0031 | 1.80E-09 | | 462346 | | 36.2 |
| rs9349379 | 6 | 12903957 | G | A | 0.4055 | | -0.0193 | 0.0031 | 3.50E-10 | | 462346 | | 39.4 |
| rs12153855 | 6 | 32074804 | C | T | 0.1050 | | 0.0294 | 0.0049 | 2.40E-09 | | 462346 | | 35.6 |
| rs9372625 | 6 | 98344031 | A | G | 0.3817 | | -0.0256 | 0.0031 | 2.90E-16 | | 462346 | | 66.9 |
| rs62466318 | 7 | 73042085 | T | C | 0.2028 | | -0.0255 | 0.0038 | 1.40E-11 | | 462346 | | 45.6 |
| rs2622167 | 7 | 153486704 | A | G | 0.4287 | | -0.0191 | 0.0031 | 4.60E-10 | | 462346 | | 38.8 |
| rs73050128 | 7 | 1961882 | A | C | 0.1645 | | -0.0260 | 0.0041 | 2.10E-10 | | 462346 | | 40.4 |
| rs6943160 | 7 | 99872071 | C | T | 0.2086 | | 0.0206 | 0.0037 | 3.10E-08 | | 462346 | | 30.6 |
| rs4726481 | 7 | 141668403 | T | G | 0.4006 | | 0.0218 | 0.0031 | 2.30E-12 | | 462346 | | 49.2 |
| rs9648478 | 7 | 39325802 | A | G | 0.5102 | | 0.0169 | 0.0030 | 2.60E-08 | | 462346 | | 31.0 |
| rs2160935 | 8 | 30840651 | T | C | 0.6043 | | -0.0187 | 0.0031 | 1.40E-09 | | 462346 | | 36.7 |
| rs34440851 | 8 | 87214346 | T | C | 0.1572 | | -0.0227 | 0.0042 | 4.60E-08 | | 462346 | | 29.9 |
| rs11787216 | 8 | 142615222 | T | C | 0.3691 | | 0.0244 | 0.0032 | 2.40E-14 | | 462346 | | 58.2 |
| rs2977454 | 8 | 141539923 | G | C | 0.1241 | | -0.0259 | 0.0046 | 1.70E-08 | | 462346 | | 31.8 |
| rs74679146 | 9 | 16287769 | C | T | 0.0745 | | -0.0321 | 0.0058 | 2.50E-08 | | 462346 | | 31.0 |
| rs489062 | 10 | 99715744 | A | G | 0.4375 | | 0.0166 | 0.0031 | 4.90E-08 | | 462346 | | 29.7 |
| rs34473884 | 10 | 133761285 | A | G | 0.2482 | | -0.0204 | 0.0035 | 6.20E-09 | | 462346 | | 33.8 |
| rs61873510 | 10 | 102626510 | T | G | 0.3279 | | 0.0204 | 0.0033 | 6.90E-10 | | 462346 | | 38.0 |
| rs4242715 | 10 | 133986135 | A | G | 0.6806 | | -0.0187 | 0.0032 | 9.30E-09 | | 462346 | | 33.0 |
| rs10792669 | 11 | 82688356 | G | A | 0.5053 | | 0.0174 | 0.0030 | 9.90E-09 | | 462346 | | 32.9 |
| rs11223617 | 11 | 133780757 | A | G | 0.2062 | | 0.0251 | 0.0038 | 2.30E-11 | | 462346 | | 44.7 |
| rs550942 | 11 | 58394154 | T | C | 0.8239 | | 0.0224 | 0.0040 | 2.00E-08 | | 462346 | | 31.5 |
| rs11039429 | 11 | 47867059 | T | C | 0.4546 | | -0.0236 | 0.0030 | 8.70E-15 | | 462346 | | 60.2 |
| rs1666658 | 11 | 121801129 | C | T | 0.3922 | | 0.0180 | 0.0031 | 6.70E-09 | | 462346 | | 33.6 |
| rs12312693 | 12 | 57511734 | C | T | 0.4518 | | -0.0177 | 0.0031 | 6.80E-09 | | 462346 | | 33.6 |
| rs7302200 | 12 | 56449435 | A | G | 0.3400 | | -0.0184 | 0.0032 | 8.40E-09 | | 462346 | | 33.2 |
| rs28768122 | 12 | 123885974 | C | T | 0.7595 | | 0.0207 | 0.0036 | 5.60E-09 | | 462346 | | 34.0 |
| rs7298932 | 12 | 23727301 | G | A | 0.1478 | | -0.0237 | 0.0043 | 3.80E-08 | | 462346 | | 30.3 |
| rs58905411 | 12 | 54623132 | A | G | 0.4101 | | -0.0266 | 0.0031 | 5.10E-18 | | 462346 | | 74.9 |
| rs1937522 | 13 | 68080817 | G | A | 0.5281 | | 0.0169 | 0.0030 | 2.50E-08 | | 462346 | | 31.1 |
| rs7330939 | 13 | 49971400 | T | C | 0.7204 | | -0.0213 | 0.0034 | 3.70E-10 | | 462346 | | 39.3 |
| rs2535911 | 14 | 73523162 | T | C | 0.3547 | | -0.0188 | 0.0032 | 2.70E-09 | | 462346 | | 35.4 |
| rs186347 | 14 | 59072226 | T | G | 0.4633 | | 0.0179 | 0.0031 | 4.00E-09 | | 462346 | | 34.6 |
| rs80292319 | 15 | 76508632 | C | T | 0.0577 | | -0.0394 | 0.0065 | 1.40E-09 | | 462346 | | 36.7 |
| rs117799466 | 15 | 34659517 | C | G | 0.3370 | | -0.0197 | 0.0033 | 3.10E-09 | | 462346 | | 35.1 |
| rs34631026 | 16 | 6172126 | T | C | 0.4461 | | -0.0169 | 0.0030 | 2.90E-08 | | 462346 | | 30.8 |
| rs72787062 | 16 | 72105844 | A | G | 0.1628 | | -0.0282 | 0.0041 | 6.40E-12 | | 462346 | | 47.2 |
| rs35105141 | 16 | 30057148 | T | C | 0.4015 | | 0.0263 | 0.0031 | 1.40E-17 | | 462346 | | 72.8 |
| rs1421085 | 16 | 53800954 | C | T | 0.4034 | | 0.0199 | 0.0031 | 1.00E-10 | | 462346 | | 41.8 |
| rs1104608 | 16 | 73912588 | C | G | 0.4263 | | 0.0174 | 0.0031 | 1.70E-08 | | 462346 | | 31.8 |
| rs8043563 | 16 | 19982353 | C | G | 0.7372 | | 0.0234 | 0.0035 | 1.70E-11 | | 462346 | | 45.3 |
| rs2411453 | 16 | 28632021 | G | T | 0.5974 | | -0.0351 | 0.0031 | 7.30E-30 | | 462346 | | 128.8 |
| rs728538 | 16 | 51205819 | G | T | 0.1689 | | 0.0229 | 0.0041 | 1.80E-08 | | 462346 | | 31.7 |
| rs9906502 | 17 | 7615745 | A | G | 0.1770 | | 0.0238 | 0.0040 | 1.90E-09 | | 462346 | | 36.1 |
| rs8614 | 17 | 27588806 | A | C | 0.1825 | | 0.0248 | 0.0039 | 2.70E-10 | | 462346 | | 39.9 |
| rs4968391 | 17 | 57780943 | T | G | 0.6749 | | -0.0193 | 0.0032 | 2.30E-09 | | 462346 | | 35.7 |
| rs9912298 | 17 | 29735752 | C | A | 0.2396 | | 0.0206 | 0.0036 | 9.70E-09 | | 462346 | | 32.9 |
| rs17690703 | 17 | 43925297 | T | C | 0.2627 | | 0.0250 | 0.0034 | 2.90E-13 | | 462346 | | 53.3 |
| rs650558 | 17 | 40721042 | T | C | 0.2479 | | 0.0207 | 0.0035 | 3.40E-09 | | 462346 | | 34.9 |
| rs1893659 | 18 | 21080859 | A | C | 0.4599 | | -0.0293 | 0.0031 | 7.60E-22 | | 462346 | | 92.3 |
| rs5022348 | 18 | 22639237 | T | C | 0.4070 | | 0.0203 | 0.0036 | 1.40E-08 | | 462346 | | 32.2 |
| rs2043677 | 18 | 38313195 | T | C | 0.1456 | | 0.0261 | 0.0043 | 1.60E-09 | | 462346 | | 36.4 |
| rs9958320 | 18 | 38269812 | C | T | 0.1531 | | 0.0249 | 0.0043 | 5.90E-09 | | 462346 | | 33.9 |
| rs62097995 | 18 | 50843233 | A | T | 0.4236 | | 0.0200 | 0.0031 | 6.90E-11 | | 462346 | | 42.5 |
| rs2924321 | 18 | 53125435 | A | G | 0.5396 | | -0.0195 | 0.0031 | 1.60E-10 | | 462346 | | 40.9 |
| rs4940926 | 18 | 57732418 | C | T | 0.7350 | | -0.0191 | 0.0034 | 2.80E-08 | | 462346 | | 30.8 |
| rs838145 | 19 | 49248730 | A | G | 0.5430 | | 0.0220 | 0.0031 | 6.70E-13 | | 462346 | | 51.6 |
| rs6030200 | 20 | 35554361 | A | G | 0.3142 | | -0.0195 | 0.0033 | 2.40E-09 | | 462346 | | 35.7 |
| rs11700855 | 21 | 34270051 | G | A | 0.0935 | | -0.0298 | 0.0052 | 1.20E-08 | | 462346 | | 32.4 |
| rs71651683 | 22 | 24828853 | T | C | 0.0142 | | -0.0705 | 0.0128 | 3.60E-08 | | 462346 | | 30.3 |
| rs1894544 | 22 | 48881562 | C | G | 0.4544 | | 0.0174 | 0.0030 | 1.10E-08 | | 462346 | | 32.6 |

* SNP, single nucleotide polymorphisms.

Supplementary Table 5. 40 SNPs associated with coffee intake.

| SNP | Chromosome | Position | Effect allele | Other allele | EAF | Beta | | Se | P | Sample size | | F | |
| --- | --- | --- | --- | --- | --- | --- | --- | --- | --- | --- | --- | --- | --- |
| rs516636 | 1 | 177855517 | A | C | 0.2089 | | 0.0117 | 0.0020 | 4.00E-09 | | 428860 | | 34.6 |
| rs4615895 | 1 | 96274668 | A | G | 0.7409 | | 0.0122 | 0.0018 | 4.20E-11 | | 428860 | | 43.5 |
| rs13387939 | 2 | 637498 | A | C | 0.8284 | | 0.0166 | 0.0021 | 9.80E-15 | | 428860 | | 59.9 |
| rs780093 | 2 | 27742603 | C | T | 0.6158 | | 0.0133 | 0.0017 | 1.00E-15 | | 428860 | | 64.4 |
| rs12989746 | 2 | 49368391 | T | G | 0.2499 | | 0.0104 | 0.0019 | 2.80E-08 | | 428860 | | 30.8 |
| rs1527961 | 2 | 62780440 | C | T | 0.1349 | | -0.0133 | 0.0024 | 1.70E-08 | | 428860 | | 31.8 |
| rs2597805 | 4 | 17424930 | T | C | 0.6825 | | 0.0099 | 0.0018 | 2.00E-08 | | 428860 | | 31.5 |
| rs2189234 | 4 | 106075498 | G | T | 0.6178 | | 0.0100 | 0.0017 | 1.80E-09 | | 428860 | | 36.2 |
| rs13163336 | 5 | 87943710 | A | C | 0.1576 | | 0.0149 | 0.0022 | 1.30E-11 | | 428860 | | 45.7 |
| rs12514566 | 5 | 7391462 | A | G | 0.3371 | | -0.0114 | 0.0017 | 2.40E-11 | | 428860 | | 44.7 |
| rs2465037 | 6 | 51179260 | A | C | 0.3430 | | -0.0106 | 0.0017 | 4.80E-10 | | 428860 | | 38.8 |
| rs1338549 | 6 | 98312143 | G | T | 0.5339 | | -0.0095 | 0.0016 | 5.60E-09 | | 428860 | | 34.0 |
| rs9398171 | 6 | 108983527 | T | C | 0.7106 | | 0.0109 | 0.0018 | 1.10E-09 | | 428860 | | 37.2 |
| rs73075167 | 7 | 17570479 | T | A | 0.1292 | | -0.0161 | 0.0024 | 5.00E-11 | | 428860 | | 43.2 |
| rs7811609 | 7 | 32930597 | T | C | 0.3747 | | 0.0091 | 0.0017 | 4.00E-08 | | 428860 | | 30.1 |
| rs1057868 | 7 | 75615006 | T | C | 0.2850 | | 0.0200 | 0.0018 | 5.40E-29 | | 428860 | | 124.9 |
| rs4410790 | 7 | 17284577 | C | T | 0.6321 | | 0.0391 | 0.0017 | 1.20E-120 | | 428860 | | 545.5 |
| rs34060476 | 7 | 73037956 | G | A | 0.1339 | | 0.0184 | 0.0024 | 7.50E-15 | | 428860 | | 60.4 |
| rs6469262 | 8 | 110443480 | C | T | 0.5650 | | -0.0092 | 0.0016 | 1.90E-08 | | 428860 | | 31.6 |
| rs78267637 | 8 | 33790200 | G | C | 0.0381 | | -0.0254 | 0.0043 | 3.90E-09 | | 428860 | | 34.7 |
| rs442355 | 8 | 109128653 | C | G | 0.2544 | | -0.0111 | 0.0019 | 1.90E-09 | | 428860 | | 36.1 |
| rs10119174 | 9 | 27953724 | C | G | 0.5710 | | -0.0094 | 0.0016 | 1.00E-08 | | 428860 | | 32.8 |
| rs117810762 | 10 | 135315795 | A | G | 0.0179 | | 0.0359 | 0.0062 | 6.20E-09 | | 428860 | | 33.8 |
| rs61928609 | 12 | 11316437 | C | A | 0.8353 | | -0.0147 | 0.0022 | 1.30E-11 | | 428860 | | 45.9 |
| rs2472297 | 15 | 75027880 | T | C | 0.2629 | | 0.0465 | 0.0018 | 1.10E-142 | | 428860 | | 646.7 |
| rs117968677 | 15 | 75174251 | A | G | 0.0242 | | -0.0310 | 0.0055 | 1.90E-08 | | 428860 | | 31.6 |
| rs8056750 | 16 | 70927078 | T | C | 0.3591 | | 0.0105 | 0.0017 | 1.30E-09 | | 428860 | | 36.8 |
| rs1421085 | 16 | 53800954 | C | T | 0.4036 | | 0.0185 | 0.0016 | 1.70E-29 | | 428860 | | 127.2 |
| rs62064918 | 17 | 46155786 | T | C | 0.2445 | | -0.0103 | 0.0019 | 4.10E-08 | | 428860 | | 30.1 |
| rs57918684 | 17 | 60150383 | A | G | 0.1547 | | 0.0129 | 0.0022 | 8.60E-09 | | 428860 | | 33.1 |
| rs7224815 | 17 | 17845800 | T | A | 0.4078 | | -0.0109 | 0.0016 | 3.70E-11 | | 428860 | | 43.8 |
| rs630194 | 18 | 40950954 | C | T | 0.3434 | | -0.0114 | 0.0017 | 2.30E-11 | | 428860 | | 44.7 |
| rs1942965 | 18 | 55032486 | C | T | 0.5046 | | -0.0089 | 0.0016 | 3.80E-08 | | 428860 | | 30.2 |
| rs476828 | 18 | 57852587 | C | T | 0.2374 | | 0.0173 | 0.0019 | 5.60E-20 | | 428860 | | 83.8 |
| rs56113850 | 19 | 41353107 | C | T | 0.5781 | | 0.0127 | 0.0016 | 8.90E-15 | | 428860 | | 60.1 |
| rs75347775 | 19 | 18495908 | A | G | 0.2445 | | 0.0105 | 0.0019 | 2.70E-08 | | 428860 | | 30.9 |
| rs6063085 | 20 | 45840459 | C | A | 0.3735 | | 0.0104 | 0.0017 | 4.50E-10 | | 428860 | | 38.9 |
| rs6062682 | 20 | 62891820 | T | C | 0.4645 | | 0.0104 | 0.0016 | 2.50E-10 | | 428860 | | 40.0 |
| rs13054099 | 22 | 41215672 | C | T | 0.2610 | | -0.0108 | 0.0018 | 4.30E-09 | | 428860 | | 34.5 |
| rs17842490 | 22 | 24870527 | G | A | 0.0142 | | -0.0452 | 0.0068 | 3.30E-11 | | 428860 | | 44.0 |

* SNP, single nucleotide polymorphisms.
